# Subthalamic Peak Beta Ratio Is Asymmetric in Glucocerebrosidase Mutation Carriers With Parkinson's Disease: A Pilot Study

**DOI:** 10.3389/fneur.2021.723476

**Published:** 2021-09-30

**Authors:** Fabian J. David, Miranda J. Munoz, Jay L. Shils, Michael W. Pauciulo, Philip T. Hale, William C. Nichols, Mitra Afshari, Sepehr Sani, Leo Verhagen Metman, Daniel M. Corcos, Gian D. Pal

**Affiliations:** ^1^Department of Physical Therapy and Human Movement Sciences, Northwestern University, Chicago, IL, United States; ^2^Department of Anesthesiology, Rush University Medical Center, Chicago, IL, United States; ^3^Division of Human Genetics, Cincinnati Children's Hospital Medical Center, Cincinnati, OH, United States; ^4^Department of Neurological Science, Rush University Medical Center, Chicago, IL, United States; ^5^Department of Neurosurgery, Rush University Medical Center, Chicago, IL, United States; ^6^Department of Neurology, Rutgers University, New Brunswick, NJ, United States

**Keywords:** GBA mutation carriers, Parkinson's disease, subthalamic nucleus beta power, deep brain stimulation, local field potential (LFP)

## Abstract

**Introduction:** Up to 27% of individuals undergoing subthalamic nucleus deep brain stimulation (STN-DBS) have a genetic form of Parkinson's disease (PD). G*lucocerebrosidase* (*GBA*) mutation carriers, compared to sporadic PD, present with a more aggressive disease, less asymmetry, and fare worse on cognitive outcomes with STN-DBS. Evaluating STN intra-operative local field potentials provide the opportunity to assess and compare symmetry between *GBA* and non-*GBA* mutation carriers with PD; thus, providing insight into genotype and STN physiology, and eligibility for and programming of STN-DBS. The purpose of this pilot study was to test differences in left and right STN resting state beta power in non-*GBA* and *GBA* mutation carriers with PD.

**Materials and Methods:** STN (left and right) resting state local field potentials were recorded intraoperatively from 4 *GBA* and 5 non-*GBA* patients with PD while off medication. Peak beta power expressed as a ratio to total beta power (peak beta ratio) was compared between STN hemispheres and groups while co-varying for age, age of disease onset, and disease severity.

**Results:** Peak beta ratio was significantly different between the left and the right STN for the *GBA* group (*p* < 0.01) but not the non-*GBA* group (*p* = 0.56) after co-varying for age, age of disease onset, and disease severity.

**Discussion:** Peak beta ratio in *GBA* mutation carriers was more asymmetric compared with non-mutation carriers and this corresponded with the degree of clinical asymmetry as measured by rating scales. This finding suggests that *GBA* mutation carriers have a physiologic signature that is distinct from that found in sporadic PD.

## Introduction

Activity from populations of neurons can be recorded in the form of local field potentials (LFPs) during deep brain stimulation (DBS) surgery for Parkinson's disease (PD) (1). The most used surgical target for PD, the subthalamic nucleus (STN), is characterized by pathologic LFP rhythms that oscillate in the low-frequency (LF) band (2–7 Hz), beta band (8–30 Hz), gamma band (60–90 Hz), and high-frequency band (>200 Hz) (2). Beta power has been a particular focus of study as it has been found to be a marker of motor impairment. In fact, the reduction in beta power after administration of levodopa is positively correlated with improvement of motor impairment (3). Similarly, continuous high frequency DBS results in a reduction in beta power and correlates with an improvement in motor function in PD (4–6).

Interestingly, relatively consistent pathologic resting state beta power characteristics have been defined for PD (1, 7) despite significant variation in PD phenotype. Though alterations in the rate and pattern of basal ganglia neurons may partly explain the phenotypic variation (8), the contribution of individual genotype to differences in beta power has not yet been explored. This is important because an increasing number of studies have demonstrated that genotype is an important predictor of DBS outcome (9–13). For instance, several studies have shown that *glucocerebrosidase* (*GBA)* mutation carriers fare worse with STN-DBS compared with non-mutation carriers from a cognitive standpoint, though they still maintain motor benefit (9–13). In contrast, *LRRK2 G2019S* mutation carriers fare the same as non-mutation carriers from a cognitive standpoint and have at least the same or even better motor outcome compared with sporadic PD patients (9–13). Very importantly, 22–27% of individuals undergoing STN-DBS have a genetic form of PD (13, 14), indicating that genetic variability could be particularly useful in understanding individual patient phenotype and DBS outcome. As LFP signals are now being used clinically in adaptive DBS systems, these signals, when combined with genotypic data, may provide important insights into genotype-phenotype relationships and the variability in DBS outcomes.

In this pilot study, we examined differences in beta power comparing *GBA* mutation carriers and non-mutation carriers with PD. We focused on *GBA* because mutation in the *GBA* gene is the most common genetic risk factor for PD and is a harbinger of aggressive cognitive and motor decline. Also, up to 17% of PD patients undergoing DBS are *GBA* mutation carriers (13, 14). In a prior study using motion analysis, our group demonstrated that *GBA* mutation carriers had more symmetric arm swing velocity compared with non-mutation carriers in the OFF state (15). Furthermore, *GBA* mutation carriers are less likely to have an asymmetric onset of PD compared with non-mutation carriers (16). Given these findings, we hypothesized that *GBA* mutation carriers would have more symmetric beta power compared with non-mutation carriers when LFPs were collected in the OFF state during STN-DBS surgery.

## Methods

### Patients

The study was approved by the Rush University Medical Center Institutional Review Board. All participants provided written informed consent for study participation. Study participants were recruited based on a convenience sample from the Rush Movement Disorder clinic between July 2016 and August 2020. All patients with PD were offered the option to participate in the study after they were judged to be candidates for bilateral STN-DBS surgery by the movement disorders surgical team and decided to undergo bilateral surgery. As part of the inclusion criteria, participants had a confirmed diagnosis of PD according to the United Kingdom Parkinson Disease Society Brain Bank criteria (17), were responsive to dopaminergic medication, had significant motor fluctuations, presented with disabling dyskinesias and/or tremor, and lacked significant cognitive impairment and dementia as determined by formal neuropsychological testing.

All subjects completed a levodopa challenge as part of their candidacy assessment for DBS. The pre-operative OFF medication motor scores are reported as the Movement Disorders Society revision of the Unified Parkinson Disease Rating Scale (MDS-UPDRS Part III) (18). In subjects with only UPDRS Part III scores, scores were converted to MDS-UDPRS Part III scores (19). The sum of the bradykinesia and rigidity items with laterality was calculated for each side of the body using the relevant UPDRS (items 22–26) and MDS-UPDRS (items 3.3–3.8) items to determine the bradykinesia-rigidity score. We considered only bradykinesia and rigidity items and excluded tremor items as beta power has been shown to be related to clinical signs of bradykinesia and rigidity and not tremor (20–22). A simple difference between left and right scores were calculated (23). Positive scores indicated left-dominant PD, negative scores indicated right-dominant PD, and a score of 0 indicated symmetric PD. Table 1 lists demographic and clinical data for the non-*GBA* and *GBA* groups.

**Table 1:** Patient characteristics, MDS-UPDRS scores, and coordinates of lead location in the left and right STN.

	**Non-*GBA* (*n* = 5)**	***GBA* (*n* = 4)**	***p*-value**
Age (years)	60.0 (7.1)	54.0 (8.5)	0.29
Disease duration (years)	9.4 (4.2)	6.3 (2.9)	0.19
Sex	M, 4; F, 1	M, 3; F, 1	
Ethnicity (n)	2	3	
Caucasian, non-Hispanic	1	1	
Caucasian, Hispanic	2	0	
Asian	0	0	
Other			
MDS-UPDRS Part III (OFF medication)	51.8 (13.6)	47.3 (8.3)	1.0
MDS-UPDRS Part III (ON medication)	22.6 (5.3)	29.8 (10.5)	0.41
Bradykinesia asymmetry score	−0.6 (3.5)	7.0 (2.9)	0.01
Left STN			
X	−10.6 (1.4)	−9.7 (1.7)	0.56
Y	−4.8 (1.0)	−4.3 (0.7)	0.41
Z	−5.0 (0.8)	−4.8 (0.9)	1.0
Right STN			
X	10.5 (1.0)	9.2 (0.2)	0.06
Y	−3.7 (1.6)	−4.4 (0.4)	0.19
Z	−6.3 (1.0)	−5.4 (1.0)	0.19

*Mean values (standard deviation) are shown; MDS-UPDRS, Movement Disorders Society Unified Parkinson's Disease Rating Scale; STN, subthalamic nucleus; GBA, Glucocerebrosidase; M, male; F, female*.

### Surgical Procedures

Participants were treated with standard clinical and surgical techniques to implant bilateral STN-DBS leads as described previously (24). Left and right STN-DBS leads were implanted during the same surgery. Participants underwent identical surgical procedures for target localization and lead implantation (i.e., stereotactic, awake, microelectrode localization, intra-operative CT, and with test stimulation to determine efficacy without adverse effects) (25, 26). Prior to the first surgery, participants underwent thin-cut high-resolution MRI brain imaging. This scan was used for direct targeting of the dorsolateral STN for DBS electrode placement, as well as co-registration with intra-operative CT to localize electrode position (26–28). Micro-electrode recording (MER) was used to assure the DBS electrode would be placed in the sensory-motor region of the STN. Confirmation that the DBS lead was in the same location as the MER tract that offered the “best” recording was done via O-arm™ (Medtronic, Inc. Minnesota, USA) spins with the micro-electrode in place and then with the DBS electrode in place. Once the leads were implanted, and before macro-stimulation testing, the LFP recording testing protocol was performed. Typically, leads were inserted, location verified, and LFPs were recorded for each side sequentially; thus, the duration between lead insertion and LFP recording was similar between the left and the right STN across participants.

### Intraoperative LFP Recording

Data were acquired on an Alpha-Omega Neuro Omega system (Alpha-Omega, Nazareth, Israel). STN LFPs were recorded from all contacts from each lead in a referential electrode configuration. The reference, a needle placed on the scalp, was placed in the skin near the surgical opening. The output from the lead was connected to a custom-made cable that transmitted information from each contact in the lead to the Alpha-Omega Neuro Omega system. Depending on the type of lead (Medtronic 3389, Boston Scientific Vercise Cartesia model DB-2202-45 or Abbott-St. Jude Infinity model 6172) that was implanted, intra-operative LFP recording resulted in 4 to 8 channels of data. Each channel corresponded to data from a single contact on the DBS lead. The data were sampled at a rate of 1,375 Hz with a gain of 55,000. Data were stored on the Neuro Omega system. Prior to transfer for further analysis, the data were converted to the MATLAB format through an Alpha-Omega conversion routine and then imported into MATLAB. LFPs from the left and right STN were recorded during rest for 120 seconds. Participants were supine on the operating table; their arms were completely supported with their elbows extended and placed at their sides. Participants were asked to relax during data collection. After obtaining a stable baseline signal for 5 seconds (limiting insertional effects), LFPs were then recorded continuously for 120 seconds. The data were visually reviewed in real time to identify signal artifact or excessive noise. Notation was also made of any tremor activity. If the motor task was interrupted or inconsistent, signal acquisition was halted and restarted. If signal artifact or excessive noise was identified, the signal acquisition was halted and restarted.

### LFP Analysis

All available channels of raw LFP data were analyzed by a rater (MJM) blinded to *GBA* status of the participants. The objective of the LFP analysis was to identify the contact with the highest beta power in the left and right STN. As beta power is known to be localized to the dorsolateral STN (29), the fundamental premise was that the contact with the highest beta power would be the most likely contact closest to the dorsolateral STN.

**Figure 1:**
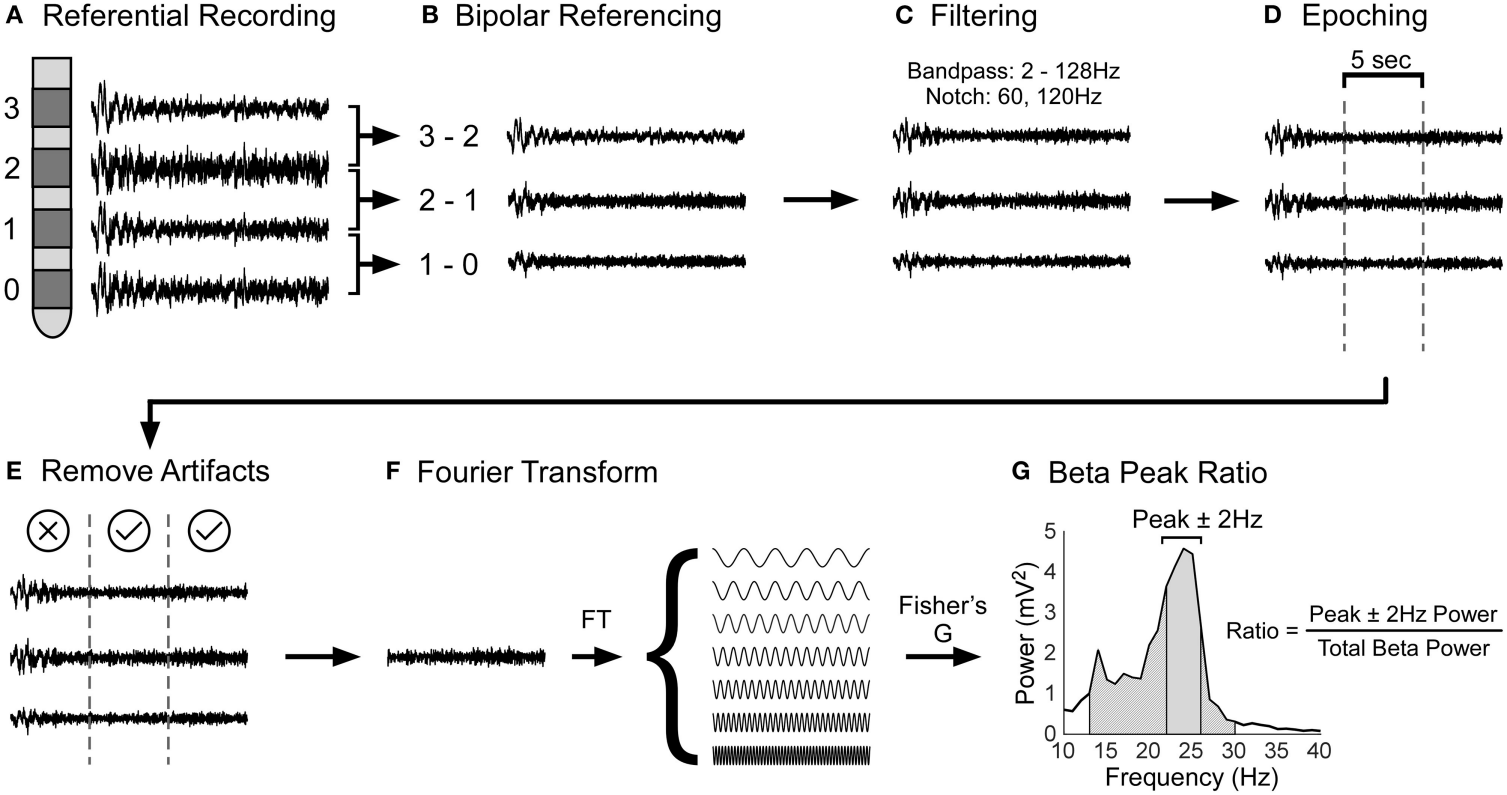
Schematic of local field potential data processing pipeline. **(A)** During surgery, STN LFPs were recorded in a referential electrode configuration. **(B)** The raw LFP data was bipolar referenced and then **(C)** filtered using a band pass (2 and 128Hz) and notch filter (60 and 120Hz). **(D)** After, the LFP data was divided into 5s epochs and **(E)** epochs with artifacts were removed from analysis. **(F)** The LFP data were Fourier transformed to determine the power of each frequency. Power in the beta band was calculated by evaluating the area under the power spectral density, which is the square of the magnitude of Fourier Transform (not shown in the figure). **(G)** Finally, the beta peak ratio was calculated by dividing the power at the frequency with peak beta ± 2Hz (solid gray segment) by the total power of the beta band (diagonally lined segment).

LFP data were processed using custom-made scripts and built-in functions of MATLAB, EEGLAB (30), and Fieldtrip (31). Figures 1A–G shows the LFP data processing pipeline. The raw LFP data was bipolar referenced (configuration for the 4-contact lead: 1–0, 2–1, 3–2; configuration for the 8-channel segmental lead: each segmental lead was referenced to the closest ring electrode) (32), band pass filtered (2 and 128 Hz), and line noise was filtered using a notch filter at 60 and 120 Hz. Then, the LFP data was divided into 5s epochs. Using EEGLAB, each 5s epoch was statistically analyzed to detect artifacts by identifying epochs with large outliers (greater than ±500 μv), abnormal linear trends (a slope >50 μv), improbable data points using a joint probability function, abnormal distribution using kurtosis of activity, and abnormal spectra (30). Each epoch statistically deemed to contain artifacts were excluded from further analysis following visual confirmation. Epochs that had tremor artifact were excluded from the analysis. Our focus was to determine the spectral power of each 5s epoch. This was carried out in FieldTrip using the ft_freqanalysis function with a Hanning window (31). Parameters used for configuring ft_freqanalysis function can be found at this link: https://www.fieldtriptoolbox.org/reference/ft_freqanalysis. A Hanning taper was used as our frequencies of interest were less than 30Hz. Our frequencies of interest were beta frequencies, i.e., 12Hz to 30Hz in steps of 1Hz. Our analysis was restricted to the beta band as the beta band has been shown to be related to bradykinesia and rigidity (20–22). Power in the beta band for each 5s epoch was calculated and averaged for each contact. Next, robust Fisher's G statistic and its accompanying *p*-value were calculated to determine if the beta peaks associated with a given contact were statistically significantly greater than surrounding peaks (33). The G-statistic is a formal test that uses the false discovery approach to determine if a given peak in a time series is statistically significantly greater than the surrounding peaks (34). Only the contact with the largest beta peak was used in subsequent analyses. For this contact, the ratio of the area under the frequency with peak beta ± 2 Hz, to the area of the entire beta band was calculated for each 5s epoch (see Figure 1G); thus, the peak beta area was normalized to the total beta area. Expressing peak beta power as a normalized value allowed us to make between subject comparisons. This peak beta ratio was calculated for the left and right STN for *GBA* and non-*GBA* participants and was used as the outcome in our statistical analysis.

### Genetic Testing

Genetic testing categorized our participants into non-*GBA* and *GBA* carriers. Enrolled participants were screened for *GBA* mutation status. Prior to STN-DBS surgery, blood samples were sent to the University of Cincinnati Biobank for molecular testing and sequenced for all *GBA* mutations as previously described (35). Study staff and subjects were blinded to mutation status during LFP recording and analysis.

### Statistical Analysis

The peak beta ratio was subject to a mixed-effects regression model. The fixed effects were Group (*GBA* and non-*GBA*), STN side (left and right), and the Group by STN side interaction. The random effect was participant. This random effect allowed for the distinction between the within-participant variance vs. the between-participant variance (i.e., it accounts for correlation within a participant), and we assumed an unstructured correlation structure. To ensure that between-group differences in age, age of onset, and disease severity (quantified by the OFF-medication MDS-UPDRS part III sub score) did not influence our results, we included these variables as covariates in our model. To test differences in left and right beta power in non-*GBA* and *GBA* mutation carriers, we performed planned pairwise comparisons (*t*-tests) on the differences of the mean least squares estimates co-varying for age, age of disease onset, and disease severity obtained from the mixed effects model. The following planned comparisons were performed: Left vs. Right STN for the non-*GBA* group, and Left vs. Right STN for the *GBA* group. All statistical tests used a two-sided 5% level of significance and *p*-values associated with pairwise comparisons were adjusted using the Bonferroni method. Normal theory methods and residual diagnostics were used to evaluate validity of assumptions. Between-group differences in demographic variables including age and disease duration, MDS-UPDRS part III sub scores, bradykinesia-rigidity asymmetry scores, and DBS lead locations were evaluated using appropriate parametric or non-parametric statistical methods. All statistical analyses were performed using SAS ® (version 9.4; SAS Institute, Cary, NC).

## Results

LFP data was obtained from 5 non-*GBA* (male, 4) and 4 *GBA* (male, 3) participants with PD. All *GBA* mutation carriers had the E326K risk variant. On average, the non-*GBA* relative to the *GBA* group: was 6 years older than the *GBA* group; had a 3.1 year longer disease duration; was 4.5 points higher on the off medication MDS-UPDRS part III sub score; was 7.2 points lower on the on medication MDS-UPDRS part III sub score; had less asymmetry and was 7 points lower on the bradykinesia-rigidity asymmetry score; was similar to the *GBA* group with respect to DBS lead locations. As can be seen in Table 1, only the bradykinesia-rigidity asymmetry score was significantly different between groups (*p* = 0.01). None of the of the other measures were statistically different between groups; however, despite the lack of statistical significance, the magnitude of difference in age (6 years), age of disease onset (3.1) and off medication MDS-UPDRS part III scores (4.5 points) may be clinically significant. Our statistical model included age, age of disease onset, and off medication MDS-UPDRS part III scores as covariates; thus, adjusting for differences between groups that may be clinically significant. The type of DBS lead and respective manufacturer are summarized in the Supplementary Table 1.

**Figure 2:**
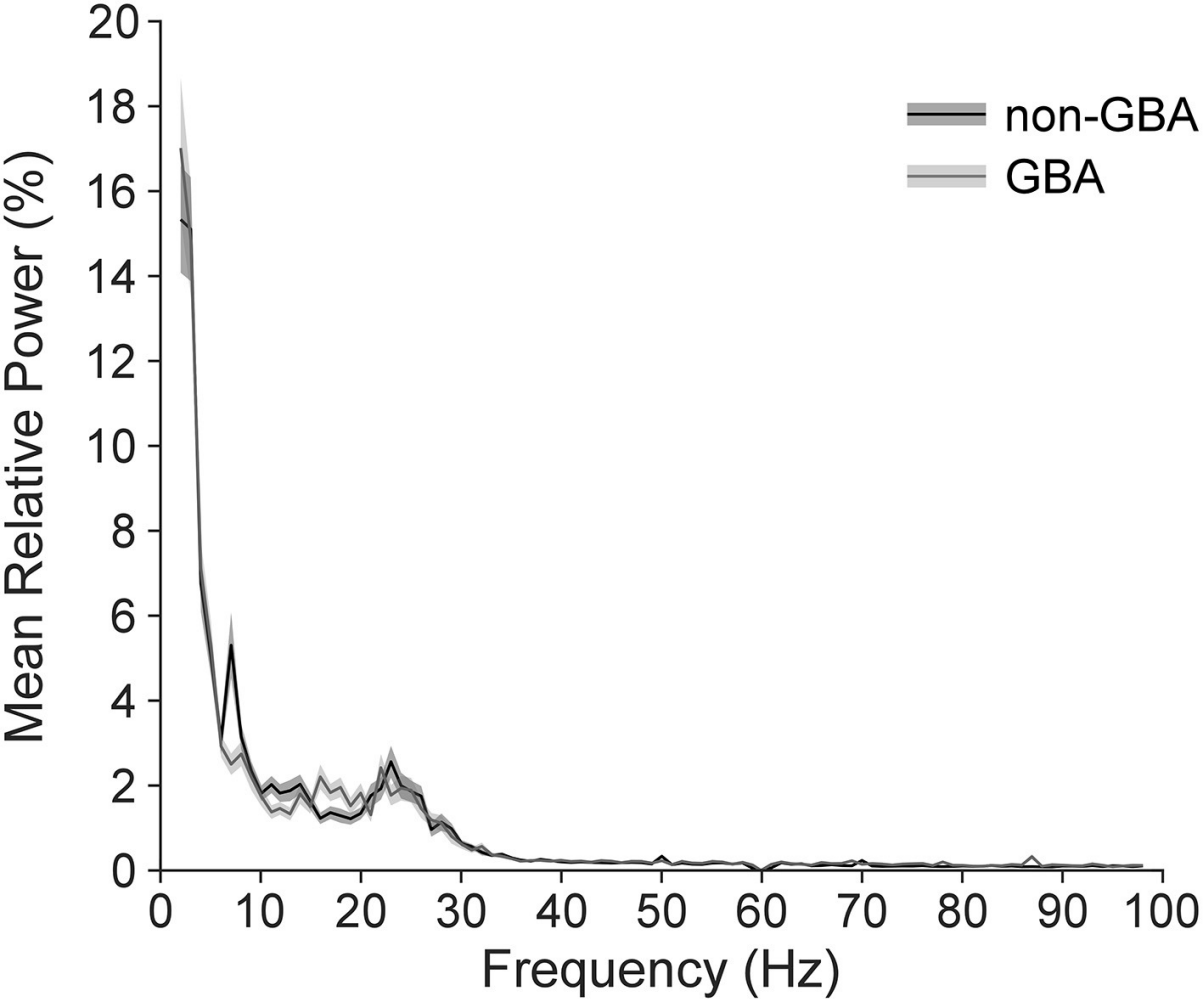
Power spectrum averaged across all participants in the non-*GBA* and *GBA* groups while off medication, estimated using the contact pairs displaying the peak beta ratio. Power in each frequency is expressed as percentage of total power in the 2–98Hz range. Note that there were no distinct peaks above 30Hz.

We observed clear beta signal in all subjects. Figure 2 illustrates the mean ± 1SE power spectrum from 2 to 98 Hz for the non-GBA and GBA groups for the contact pair displaying the maximum beta ratio. Power in each frequency was represented as a percentage of total power in the 2–98 Hz range. Figure 2 shows a marked increase in the theta band (4–8 Hz) in the non-GBA group compared to the GBA group, distinct beta peaks in both the non-GBA and GBA groups, and a lack of distinct peaks beyond 30 Hz.

**Figure 3:**
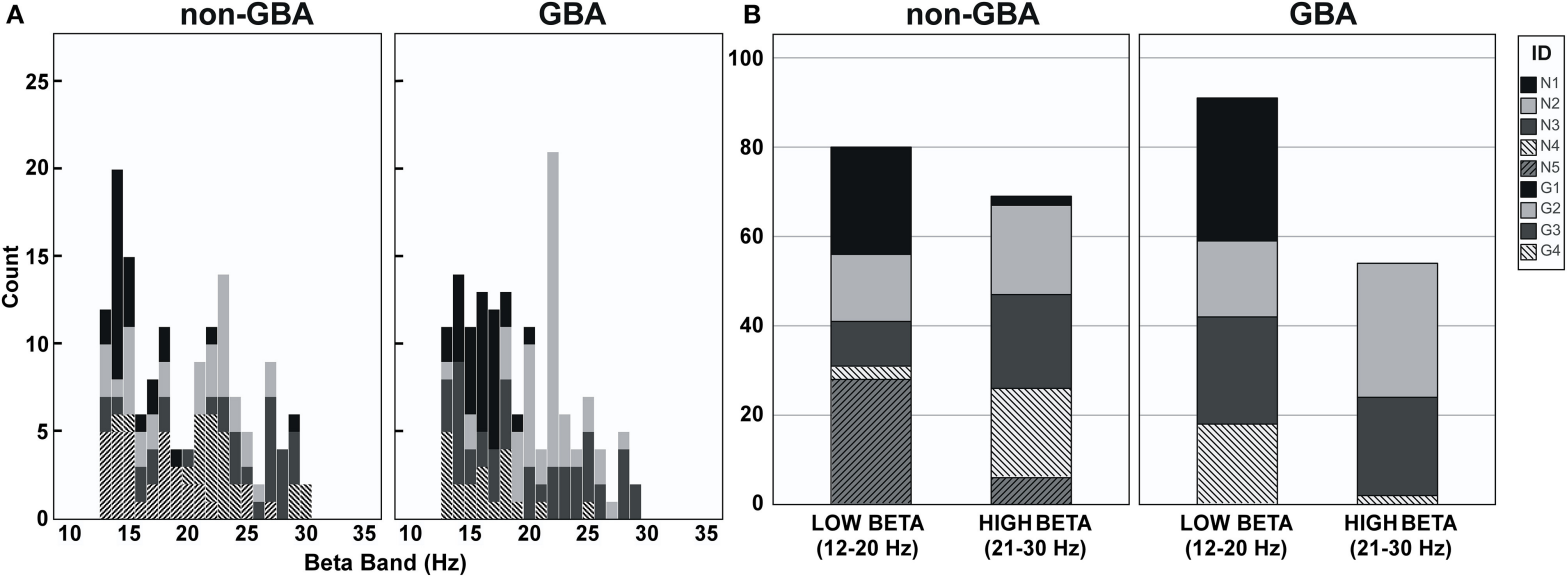
**(A)** Histogram of peak frequencies in the beta band for participants in the non-GBA and GBA groups. **(B)** Histogram of peak frequencies in the low beta band (12–20Hz) and the high beta band (21–30Hz) for participant in the non-GBA and GBA groups.

Figure 3A is a histogram illustrating the distribution of the frequency of beta peaks by participant in the non-GBA and GBA groups. As can be seen in Figure 3A, the beta peak frequencies are quite variable between the non-GBA and GBA groups, as well as between subjects within each group. Figure 3B is a histogram of peaks in the low beta (12–20Hz) and the high beta (20–30 Hz) bandwidth. There were no differences with respect to the peak beta frequencies between groups.

**Figure 4:**
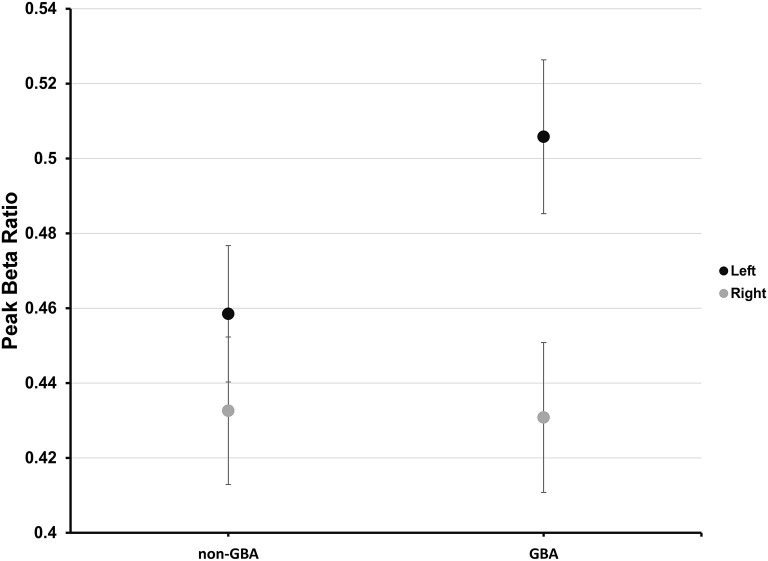
Estimated mean peak beta ratio in the non-*GBA* and *GBA* groups for the left (black circles) and right (gray circles) STN after co-varying for age, age of onset of PD, and disease severity. The error bars reflect ±1 standard error.

Planned pairwise comparisons revealed that peak beta ratio between the left and right STN was similar for the non-*GBA* group (estimated difference, 0.03; 95% confidence level, −0.03 to 0.08; *p* = 0.56; Figure 4) but was significantly different for the *GBA* group (estimated difference, 0.08; 95% confidence level, 0.02 to 0.13; *p* < 0.01; Figure 4). The degree of beta asymmetry corresponded with the degree of clinical asymmetry as measured by the bradykinesia-rigidity asymmetry score (Table 1).

**Figure 5:**
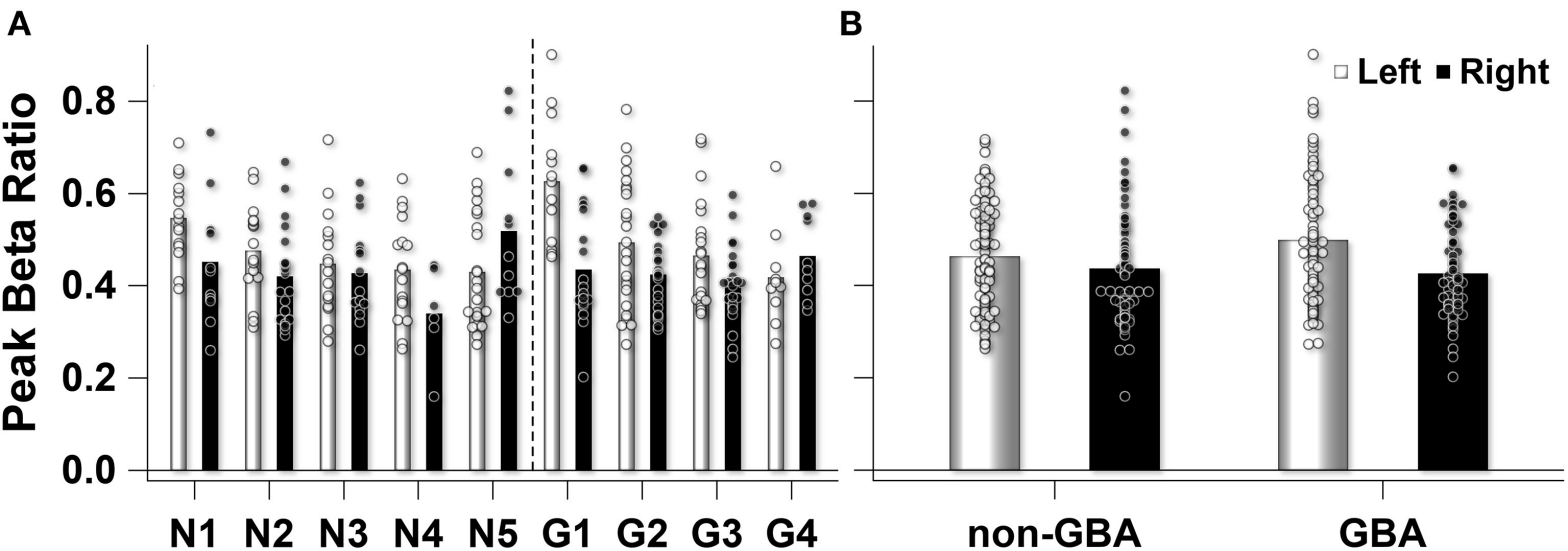
**(A)** Observed peak beta ratio for each participant in the non-*GBA* (N) and *GBA* (G) group. The unfilled (left STN) and filled (right STN) bars show the mean peak beta ratio for each participant. The unfilled (left STN) and filled (right STN) circles represent the peak beta ratio for each 5s epoch that contributed to the mean for each participant. **(B)** The mean peak beta ratio for the non-*GBA* (*n* = 5) and *GBA* (*n* = 4) participants. Unfilled bars and circles represent left STN mean and individual 5s epochs that contributed to the mean, while the filled bars and circles represent the right STN.

Participant level data demonstrated that the number of 5s epochs that contributed to the participant mean peak beta ratios were similar between participants in the non-*GBA* (145 epochs) and *GBA* (149 epochs) groups (Figure 5A). One participant in the non-GBA contributed only 6 epochs for the right STN, else the number of epochs were similar between groups. Supplementary Table 2 lists the number of number of epochs used for each participant. A Mann-Whitney U test confirmed that there were no differences between groups (*p* = 0.46). In addition, as can be seen in Figure 5A there was considerable within-participant variability in the peak beak beta ratio (estimate, 0.014; Wald Z, 11.92; *p* < 0.001), but the between-participant variability was similar (0.0005, Wald Z, 1.13; *p* = 0.13). Figure 5B summarizes the data presented in Figure 5A.

## Discussion

In this LFP pilot study, the peak beta ratio in *GBA* mutation carriers with PD was more asymmetric compared with non-mutation carriers and this corresponded to the degree of clinical asymmetry as measured by the bradykinesia-rigidity asymmetry score. This finding demonstrates that *GBA* mutation carriers may have a physiologic signature that is distinct from that found in sporadic PD. We also observed a marked increase in theta band activity in the non-GBA group compared to the GBA group. We recognize that this may be an important distinguishing feature between non-GBA and GBA participants with PD and requires further research.

Our results are consistent with a study by McNeill et al. (2013) which demonstrated that *GBA* mutation carriers with PD had greater asymmetry of radio-ligand uptake on DATscan imaging compared with other genetic forms of PD (36). Penetrance of *GBA* is only 10% at 60 years of age and 19% by 80 years of age (37), indicating that mutations in the gene are not sufficient to induce neurodegeneration. Therefore, in *GBA* mutation carriers, conversion to PD may be due to the combination of asymmetric focal neurodegeneration (related to the abnormal GCase activity) that is then exacerbated by other factors (e.g., head trauma, environmental toxins, etc.). The rate of neurodegeneration is accelerated in *GBA* mutation carriers compared with sporadic PD (38), and thus it would be reasonable to expect LFP signal asymmetry in *GBA* mutation carriers as demonstrated in the present study. As mutations in the GCase enzyme result in accumulation of sphingolipids and subsequent alpha-synuclein accumulation (39), the process by which this translates to changes in beta is unknown.

The greater asymmetry found in *GBA* mutation carriers in this study is seemingly at odds with our prior study in which we found *GBA* mutation carriers with PD demonstrated more *symmetric* arm swing velocity compared with non-mutation carriers in the OFF state, while arm range of motion and stride length were not different between the two groups (15). This may be because sensor-based motion analysis provides more granular detail regarding measurements of motor function, deconstructing bradykinesia into variety of specific parameters such as arm swing velocity, arm range of motion, and stride length. In contrast, LFPs provide information about the physiologic state of the brain and the contralateral body at rest (as does DATscan), but only to the extent that the motor region of the STN is traversed by the lead. If the DBS electrode is in the homuncular region that comprises primarily the head and arm region of the STN, LFPs from these regions will be overrepresented compared to the lower extremity. Given the small size of the STN, typically on the order of 180 mm^3^ (40), to our knowledge it is not possible to study the physiological correlate of one specific region of the body in pure isolation from neighboring body regions. Another possibility is that our finding is specific to our sample: the *GBA* group was simply more asymmetric than the non-*GBA* group; both with respect to beta power and bradykinesia-rigidity asymmetry scores. In addition, subjects in the gait study were walking and not at rest as in the present study, thereby limiting the utility of comparison of the two studies. Finally, we did not take into account the influence of specific phenotypes on changes to beta power, i.e. tremor dominant PD vs. postural instability gait disorder phenotypes. Godinho et al. (2021) found that supervised learning algorithms aimed at discriminating PD phenotypes based on STN-LFP band power features were most accurate when tremor dominant and postural instability gait disorder movement-induced desynchronization ranges were considered (41). Future studies can employ such algorithms and include genotype to improve phenotypic classification using physiologic signals.

The strengths of this study include a population of *GBA* mutation carriers with the same mutation, E326K, the length of LFP recording time, and consistency of results across participants. Limitations include the small sample size and lack of inclusion of subjects with mild or severe *GBA* mutations. With the recent FDA approval of the Medtronic Percept™ PC (42), that can record LFPs using its BrainSense™ technology, studies such as this will be able to be performed and reproduced in the clinic rather than the operating room, and additional differences, particularly related to cognition, can be examined in *GBA* mutation and non-mutation carriers. Furthermore, genetic testing for PD, especially pre-DBS, is not the current standard of care. There is increased interest in utilizing genetics to understand outcomes of interventions such as DBS (43). As the incorporation of genetic testing into the clinical setting becomes routine and as we gain access to LFPs outside of the operating room, studies such as this can be performed with more facility and larger samples sizes. Lastly, we acknowledge the clinical differences between the groups as a limitation. The non-GBA group was on average 6 years older, had 3 more years disease duration and this may result in less asymmetry. However, *GBA* carriers have faster motor progression as than their non-GBA counterparts (38) and may come to DBS earlier, so it is difficult to compare individuals with similar disease durations. In our sample, there was no correlation between disease duration and beta power. However, we cannot exclude that the non-GBA group could have presented with less asymmetry just because they had a longer disease duration. Furthermore, we used age, age of onset, and disease severity as covariates in our statistical model to adjust for these differences between groups.

The results of this study requires verification in a larger cohort, and future studies should determine if the asymmetry found in *GBA* mutation carriers correlates with mutation severity. Critically, given that that the electrophysiological characteristics of LFP-STN recordings are unknown for GBA carriers, future studies should conduct a broader electrophysiological analysis. This analysis should include a larger sample size such that it has sufficient power to identify group differences in the theta, alpha, and gamma bands, as well as differences in beta features such as beta burst duration, strength, and frequency. As LFP recordings are being used to develop long-term feedback control signals for adaptive DBS systems, genotype-phenotype studies such as this one may be useful in understanding pattern variations of LFP signals that can be used to refine or improve such systems.

## Data Availability Statement

The statistical databases supporting the conclusions of this article will be made available by the corresponding author, Fabian J. David, upon reasonable request. Due to the nature of this research, participants of this study did not agree for their data to be shared publicly, so raw data is not available.

## Ethics Statement

The studies involving human participants were reviewed and approved by Rush University Medical Center Institutional Review Board. The patients/participants provided their written informed consent to participate in this study.

## Author Contributions

Research project: JS and GP: conception. FD, MM, JS, SS, and GP: organization. FD, MM, JS, MP, PH, WN, MA, SS, LV, and GP: execution. Statistical analysis: FD: design and execution. MM, JS, LV, DC, and GP: review and critique. Manuscript: FD and GP: writing of the first draft. MM, JS, MP, PH, WN, MA, SS, LV, and DC: review and critique. All authors contributed to the article and approved the submitted version.

## Funding

National Institute of Neurological Disorders and Stroke (K23-NS097625, R01NS092950, T32 NS047987, and F31 NS120695), and the Parkinson's Foundation

## Conflict of Interest

FD and MM received grant support from NIH. JS receives consulting fees from Medtronic. SS received grant support from NIH, Medtronic, Abbott, and Boston Scientific. LV has research support from Michael J. Fox Foundation, Medtronic, Inc., US WorldMeds LLC, Pfizer Inc, Boston Scientific, Avanir Pharmaceuticals, Inc., and Adamas Pharmaceuticals, Inc.; is on the scientific advisory board of Britannia Pharmaceuticals Ltd.; and consults for Medtronic, Inc., and Boston Scientific. DC received grant support from NIH and Michael J. Fox and receives lecture and reviewer fees from NIH. GP received grant support from NIH and the Parkinson's Disease Foundation. The remaining authors declare that the research was conducted in the absence of any commercial or financial relationships that could be construed as a potential conflict of interest.

## Publisher's Note

All claims expressed in this article are solely those of the authors and do not necessarily represent those of their affiliated organizations, or those of the publisher, the editors and the reviewers. Any product that may be evaluated in this article, or claim that may be made by its manufacturer, is not guaranteed or endorsed by the publisher.
