# Supplementary material for: Subthalamic Peak Beta Ratio Is Asymmetric in Glucocerebrosidase Mutation Carriers With Parkinson's Disease: A Pilot Study
Source: Front Neurol. 2021 Sep 30;12:723476. doi: 10.3389/fneur.2021.723476 (PMC8514636; doi:10.3389/fneur.2021.723476)
Supplement: Supplementary file 1 [file Table_1.docx]

Supplementary Table 1. Lead types and manufacturers

|  | Non-*GBA*  (n=5) | *GBA*  (n=4) |
| --- | --- | --- |
| Segmented lead (n, %) | 3 (60%) | 2 (50%) |
| Non-segmented lead (n, %) | 2 (40%) | 2 (50%) |
| Manufacturer (n) |  |  |
| Medtronic | 2 | 2 |
| Boston Scientific | 1 | 1 |
| St. Jude-Abbott | 2 | 1 |

Supplementary Table 2. Number of epochs used for each participant

| ID | STN Side | |
| --- | --- | --- |
|  | Left | Right |
| N1 | 15 | 11 |
| N2 | 16 | 19 |
| N3 | 16 | 15 |
| N4 | 17 | 6 |
| N5 | 23 | 11 |
| G1 | 13 | 19 |
| G2 | 24 | 23 |
| G3 | 22 | 24 |
| G4 | 10 | 10 |

N, non-*GBA;* G*, GBA*
